# Linear systems with unstructured multiplicative uncertainty: Modeling and robust stability analysis

**DOI:** 10.1371/journal.pone.0181078

**Published:** 2017-07-12

**Authors:** Radek Matušů, Bilal Şenol, Celaleddin Yeroğlu

**Affiliations:** 1 Centre for Security, Information and Advanced Technologies (CEBIA–Tech), Faculty of Applied Informatics, Tomas Bata University in Zlín, Zlín, Czech Republic; 2 Department of Computer Engineering, Faculty of Engineering, Inonu University, Malatya, Turkey; Chongqing University, CHINA

## Abstract

This article deals with continuous-time Linear Time-Invariant (LTI) Single-Input Single-Output (SISO) systems affected by unstructured multiplicative uncertainty. More specifically, its aim is to present an approach to the construction of uncertain models based on the appropriate selection of a nominal system and a weight function and to apply the fundamentals of robust stability investigation for considered sort of systems. The initial theoretical parts are followed by three extensive illustrative examples in which the first order time-delay, second order and third order plants with parametric uncertainty are modeled as systems with unstructured multiplicative uncertainty and subsequently, the robust stability of selected feedback loops containing constructed models and chosen controllers is analyzed and obtained results are discussed.

## 1. Introduction

The robustness of control systems represents an attractive research topic whose necessity is boosted by everyday control engineering practice [1–5]. The principal problem is that the mathematical model of a controlled plant almost never matches real plant behavior exactly due to the understandable effort to build and use a simple enough LTI model in which the potential complex features are simplified or neglected and furthermore, since the real physical parameters of a system can vary owing to a number of reasons. The popular and effective approach as to how to systematically study the influence of uncertainty and overcome this discrepancy in control tasks is provided by robust control. The problem of designing a robust controller typically means ensuring the robust stability and robust performance [6–8].

Basically, uncertainty in LTI SISO systems can be taken into consideration in two main ways. Either one can use a model with parametric uncertainty [9–13] whose structure is fixed but the parameters are assumed to lie within given bounds or a model with unstructured uncertainty [14–19] where even an unknown order can be used. Both methods have their pros and cons. Parametric uncertainty seems to be more natural and relatively simple to understand while the advantage of unstructured uncertainty is generally easier application of sophisticated controller design methods (e.g. based on H_∞_) [20]. Alternatively, a mixture of parametric and unstructured uncertainty is studied in [21].

The powerful tool for (not only) uncertainty description in LTI Multiple-Input Multiple-Output (MIMO) systems is represented by the Linear Fractional Transformations (LFTs) [22]. The LFT approach allows incorporating various kind of perturbations into MIMO models with so-called structured uncertainty by means of a block-diagonal matrix of (typically stable) perturbations. The structured singular value [22, 23] based technique for the case of mixed parametric uncertainty and unmodeled dynamics is presented e.g. in [24]. On the other hand, the unstructured uncertainty for MIMO systems can be defined by use of a “full” complex perturbation matrix [14].

Be reminded that all variations in studied sort of systems are supposed to occur only “slowly” as the LTI systems are considered. Some results on the robust stability of time-variant systems can be found e.g. in [25–27].

This article deals with one kind of unstructured uncertainty, known as the multiplicative uncertainty. Its main aim is to present an approach to construct a multiplicative uncertainty model from an LTI SISO system with real parametric uncertainty and also to depict a technique for robust stability analysis. Within the scope of the presented illustrative examples, the first order time-delay, second order and third order plants with parametric uncertainty are modeled as systems with unstructured multiplicative uncertainty (by means of the suitable choice of a nominal system and a weight function), and sequentially, the robust stability of selected feedback loops with obtained plant models and several controllers is investigated. The primary technical contribution of this paper lies in its survey of several specific techniques for the construction of unstructured multiplicative uncertainty models, with the emphasis on the frequently used transfer functions of the controlled plants. Moreover, the work discusses the conservatism in the robust stability tests of the feedback control loops where the “original” parametric uncertainty plant is replaced by an unstructured multiplicative uncertainty model. Some preliminary results related to this paper and a comparison of parametric and unstructured approaches to uncertainty modeling can be found in [18, 19].

The article is organized as follows. In Section 2, uncertainty modeling techniques with a special accent on unstructured multiplicative uncertainty are briefly described. Section 3 then provides the fundamentals of robust stability analysis for the class of systems being studied. Next, the set of three comprehensive examples (for first order time-delay, second order, and third order plant) focused on modeling and robust stability analysis is presented in the extensive Section 4. And finally, Section 5 offers some concluding remarks.

A preliminary version of this article was presented at the 19th International Conference on Systems, Zakynthos, Greece, 2015 [28].

## 2. Modeling of uncertainty

The first and fundamental step in robust control is to respect the difference between the true behavior of a control loop and its mathematical description by means of exploiting the uncertain model. Roughly speaking, one fixed “nominal” model is replaced by a whole family of models represented by some neighborhood of the nominal one. This neighborhood can be quantified essentially by means of two main approaches.

The first technique, using parametric uncertainty, supposes known structure of the system (known order), but the imprecisely known real physical parameters. In practice, the parametric uncertainty is given through intervals which bound the uncertain parameters. For details, see e.g. [9–13].

On the other hand, the second, unstructured uncertainty approach does not even need any knowledge of the model structure and its description grounds in restriction of the frequency characteristics spread [14–19].

The parametric uncertainty approach is natural and advantageous from the viewpoint of its relative simplicity, while the unstructured uncertainty approach is favorable especially for unmodeled dynamics or nonlinearities and, furthermore, preferential for H_∞_ control design methods. Furthermore, various mixtures of uncertainties were investigated e.g. in [21, 24].

One can distinguish among several types of unstructured uncertainty models, i.e. multiplicative and additive models and their inverse versions, which allow describing also the unstable dynamics [14]. This paper deals with probably the most frequently utilized multiplicative model which can be described by:

$$G(s)=\left[1+W_{M}(s)\Delta_{M}(s)\right]G_{0}(s)$$ (1)

where *G*(*s*) represents an uncertain (perturbed) model, *G*_0_(*s*) is a nominal model, *W*_*M*_(*s*) means a stable weight function representing uncertainty dynamics (i.e. the distribution of the maximum magnitude of the uncertainty over the frequency), and Δ_*M*_(*s*) stands for the uncertainty itself (uncertain information on the actual magnitude and phase of perturbation), which can be represented by an arbitrary stable function fulfilling the inequality:

$${\left\|\Delta_{M}(s)\right\|}_{\infty }\leq 1\Rightarrow \left|\Delta_{M}(j\omega )\right|\leq 1\;\forall \omega$$ (2)

**Fig 1:**
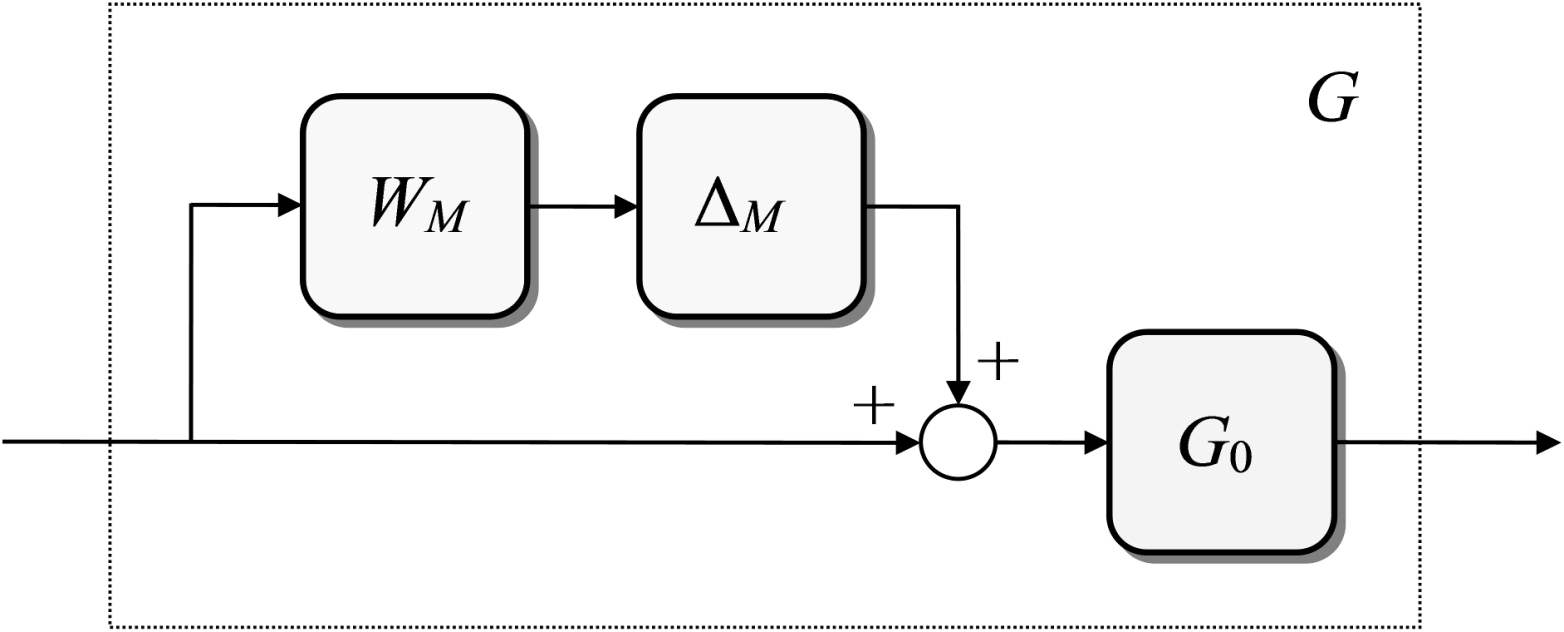
A system with multiplicative uncertainty.

The scheme of the multiplicative uncertainty (1) is depicted in Fig 1.

The multiplicative uncertainty has formally two variants, i.e. the input and output one. The input version corresponds to Fig 1 and Eq (1) while the output version has reversed order of the nominal model block and the parallel branch with a weight and uncertainty, that is:

$$G(s)=G_{0}(s)\left[1+W_{M}(s)\Delta_{M}(s)\right]$$ (3)

Obviously, both versions are equivalent for SISO case, but they have to be distinguished for MIMO systems. The analogical structure holds true also for the inverse multiplicative uncertainty.

The choice of a suitable weight function is the important part of the model creation as will be shown in the examples hereinafter. For the weight function, the following inequality, where the left-hand side represents normalized perturbation (relative error), must be fulfilled:

$$\left|\frac{G(j\omega )}{G_{0}(j\omega )}-1\right|\leq \left|W_{M}(j\omega )\right|\;\forall \omega$$ (4)

Many theoretical tools presume that all members of the family *G*(*s*) have the same amount of right-hand (unstable) poles. In other words, that *G*(*s*) and *G*_0_(*s*) have the same amount of right-hand poles for all Δ_*M*_(*s*) [14].

## 3. Robust stability analysis

Under the assumption of multiplicative uncertainty, the closed-loop system is robustly stable if and only if [14, 15]:

$${\left\|W_{M}(s)T_{0}(s)\right\|}_{\infty }<1$$ (5)

where *T*_0_(*s*) represents a complementary sensitivity function defined by:

$$T_{0}(s)=\frac{L_{0}(s)}{1+L_{0}(s)}$$ (6)

and where *L*_0_(*s*) is the open-loop frequency transfer function:

$$L_{0}(s)=C(s)G_{0}(s)$$ (7)

From the fundamental inequality (5), the following relation can be derived:

$$\left|\frac{W_{M}(j\omega )L_{0}(j\omega )}{1+L_{0}(j\omega )}\right|<1\;\forall \omega \Rightarrow \left|W_{M}(j\omega )L_{0}(j\omega )\right|<\left|L_{0}(j\omega )-\left(-1\right)\right|\;\forall \omega$$ (8)

**Fig 2:**
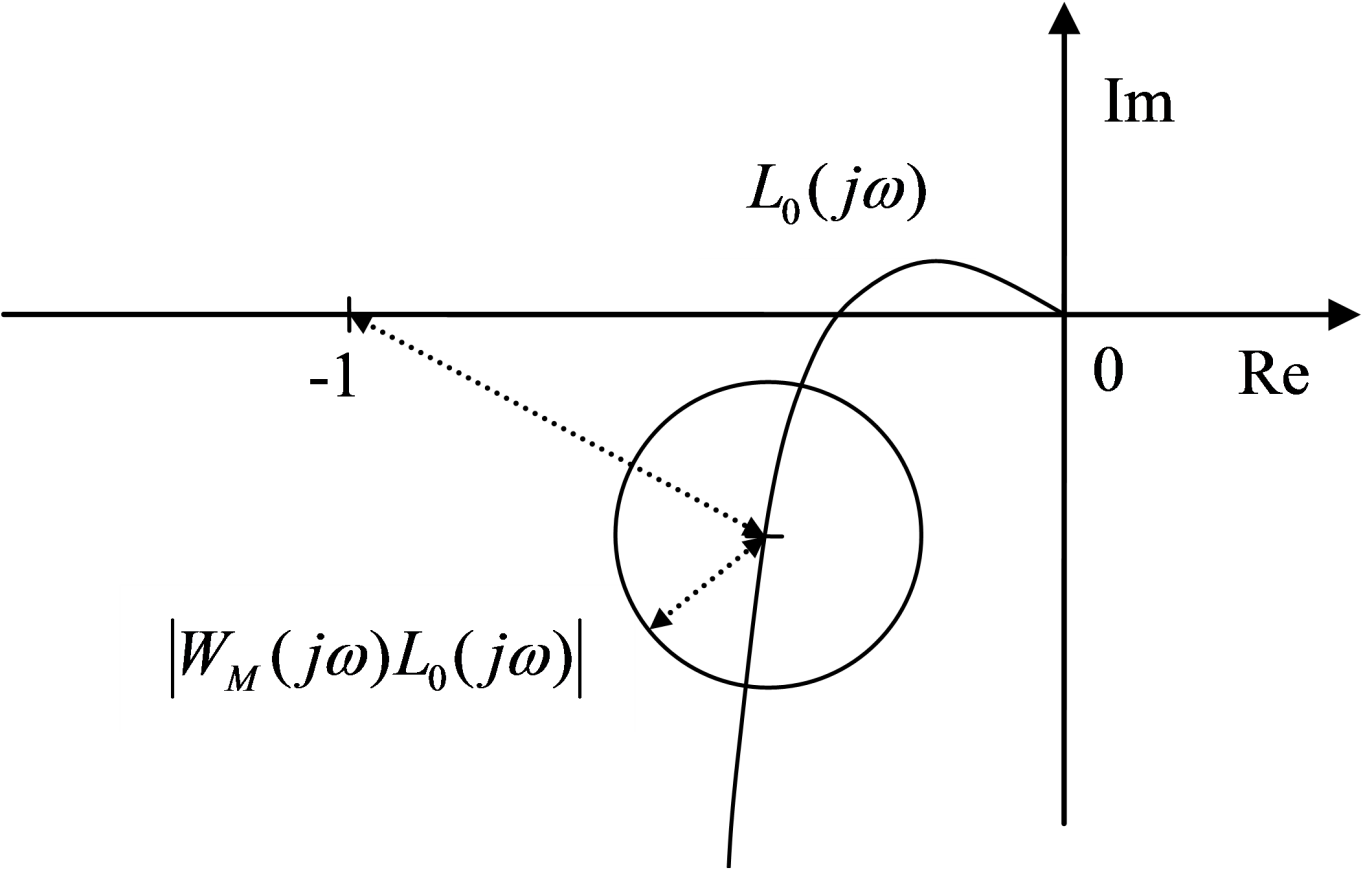
Graphical interpretation of the robust stability condition for multiplicative uncertainty.

This expression means that the closed-loop system is robustly stable if and only if the envelope of Nyquist diagrams with a radius of |*W*_*M*_(*jω*)*L*_0_(*jω*)| and center *L*_0_(*jω*) does not include the critical point [-1, 0*j*]. The depiction of this condition is shown in Fig 2.

Furthermore, the robust stability condition (5) can also have this alternative formulation:

$$\left|T_{0}(j\omega )\right|<\frac{1}{\left|W_{M}(j\omega )\right|}\;\forall \omega$$ (9)

which expresses an upper bound restriction on complementary sensitivity.

## 4. Illustrative examples–modeling and analysis

This key section presents examples of the possible construction of multiplicative uncertainty model for the commonly used forms of controlled plants, i.e. for the first order time-delay plant, second order plant and third order plant. Moreover, robust stability is analyzed for a closed loop with the second or third order plant model and selected feedback controllers.

**Fig 3:**
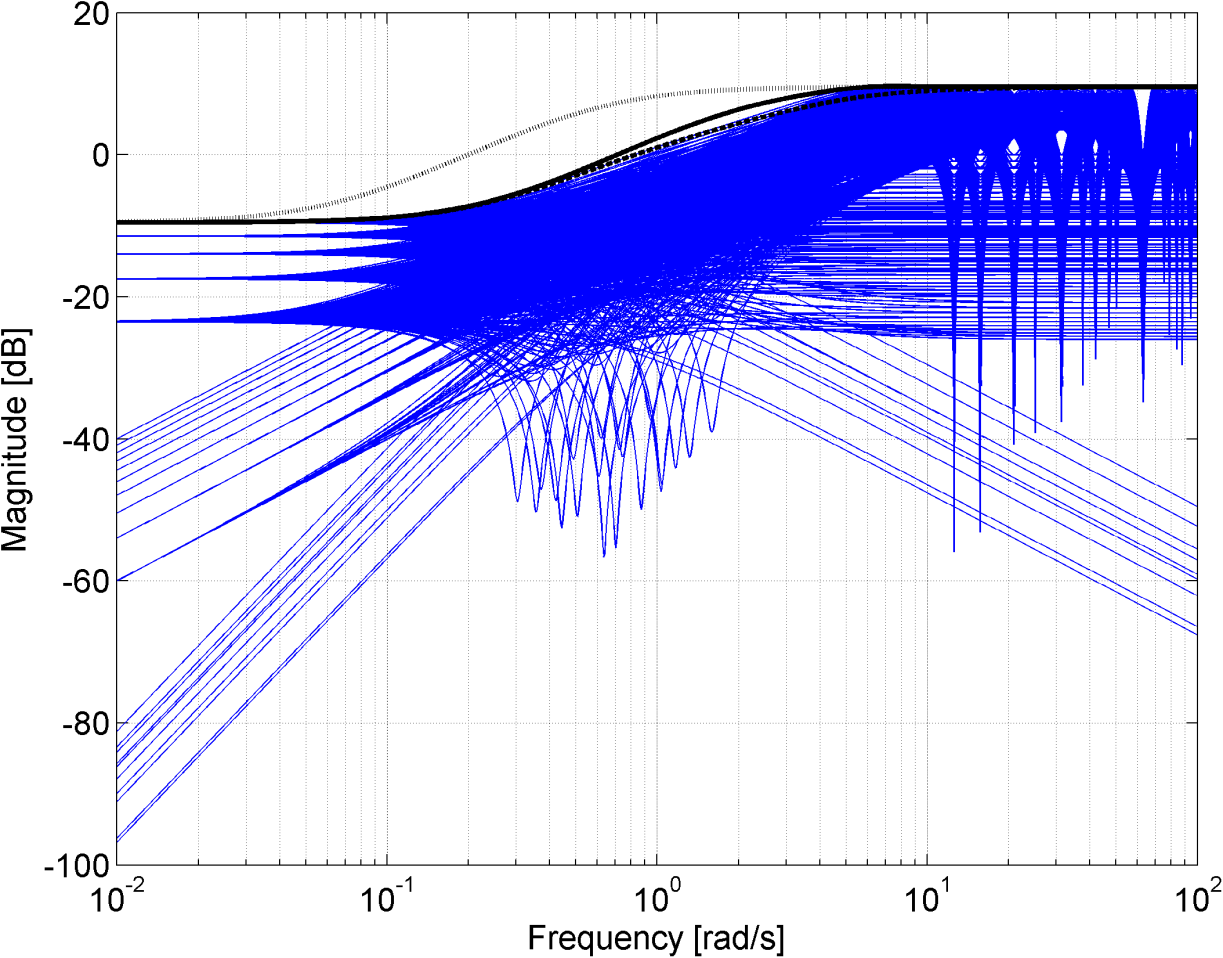
Bode magnitude plots–the set of normalized perturbations and weight functions W_M1_ (13) (dashed line), W_M2_ (15) (dotted line) and W_M3_ (16) (solid line).

**Fig 4:**
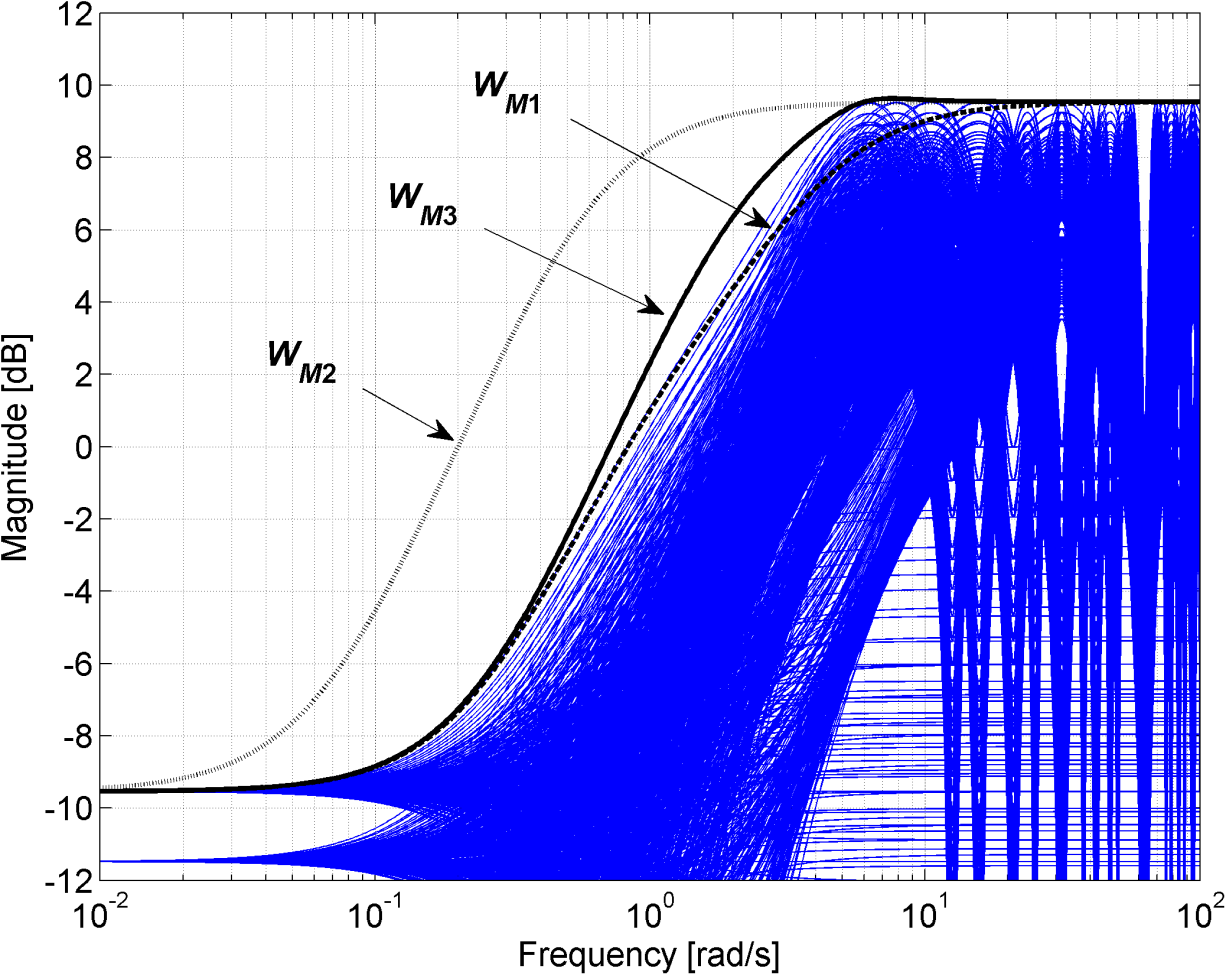
Zoomed version of Bode magnitude plots from Fig 3 –the set of normalized perturbations and weight functions W_M1_ (13) (dashed line), W_M2_ (15) (dotted line) and W_M3_ (16) (solid line).

**Fig 5:**
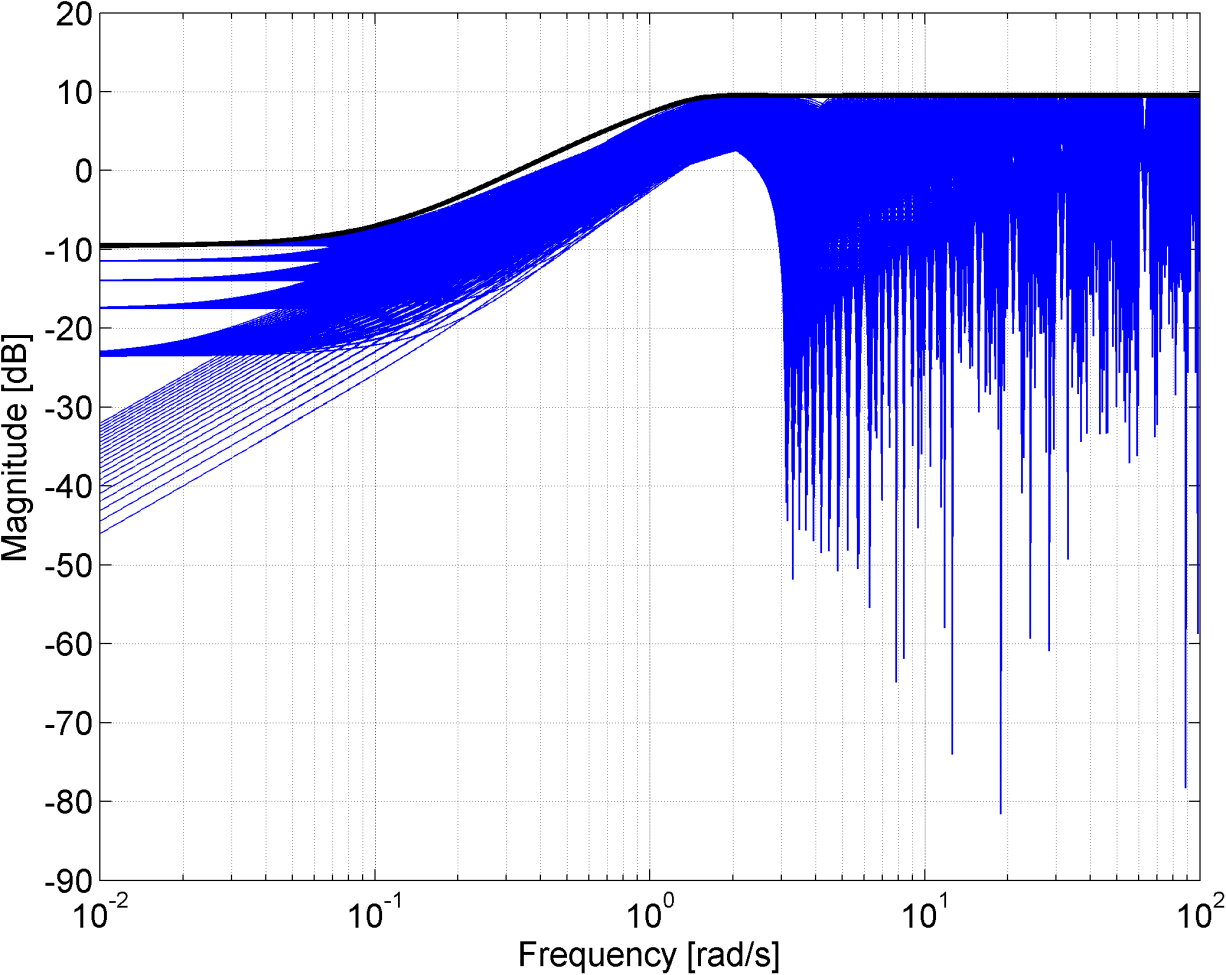
Bode magnitude plots–the set of normalized perturbations and weight function W_M4_ (18).

**Fig 6:**
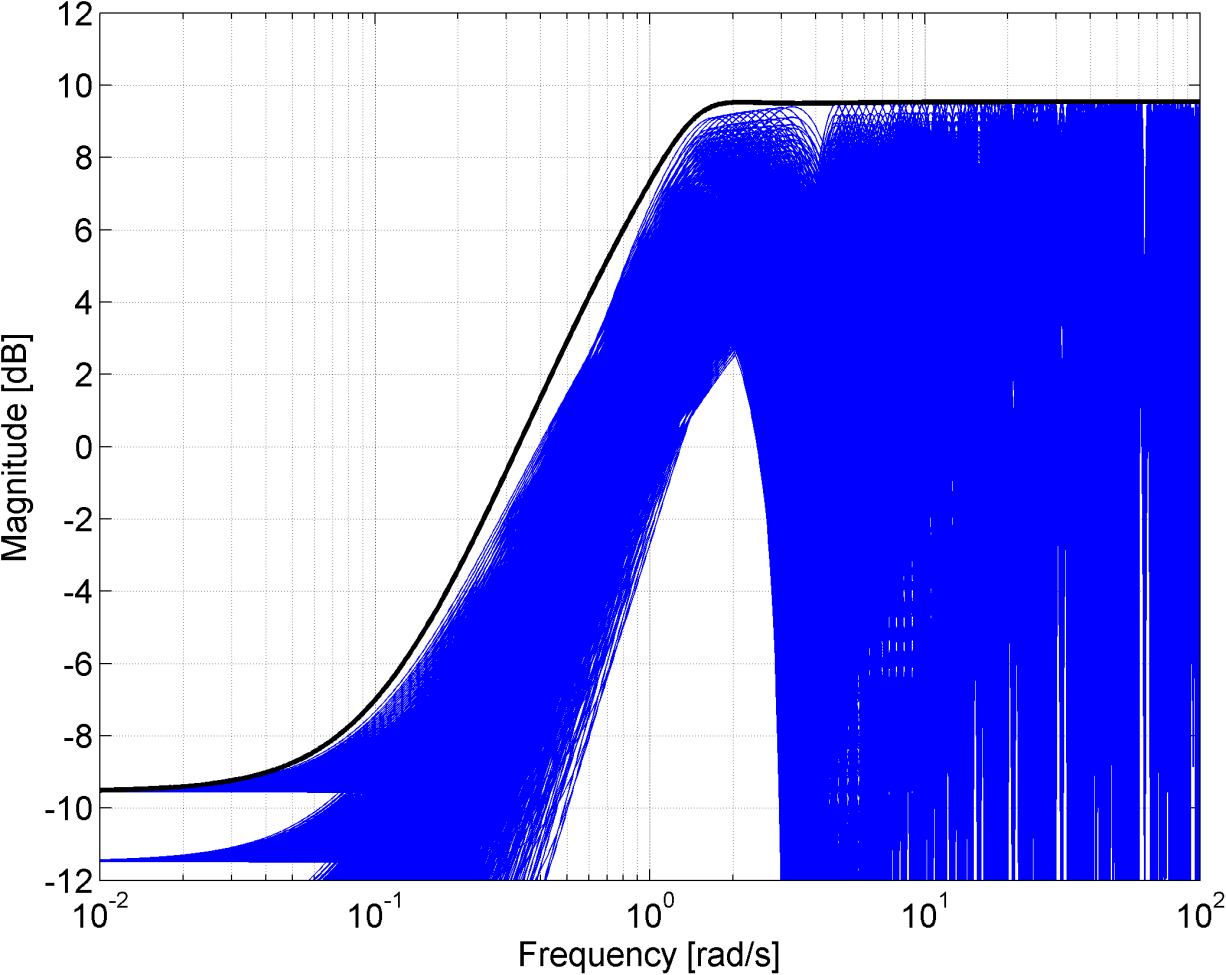
Zoomed version of Bode magnitude plots from Fig 5 –the set of normalized perturbations and weight function W_M4_ (18).

**Fig 7:**
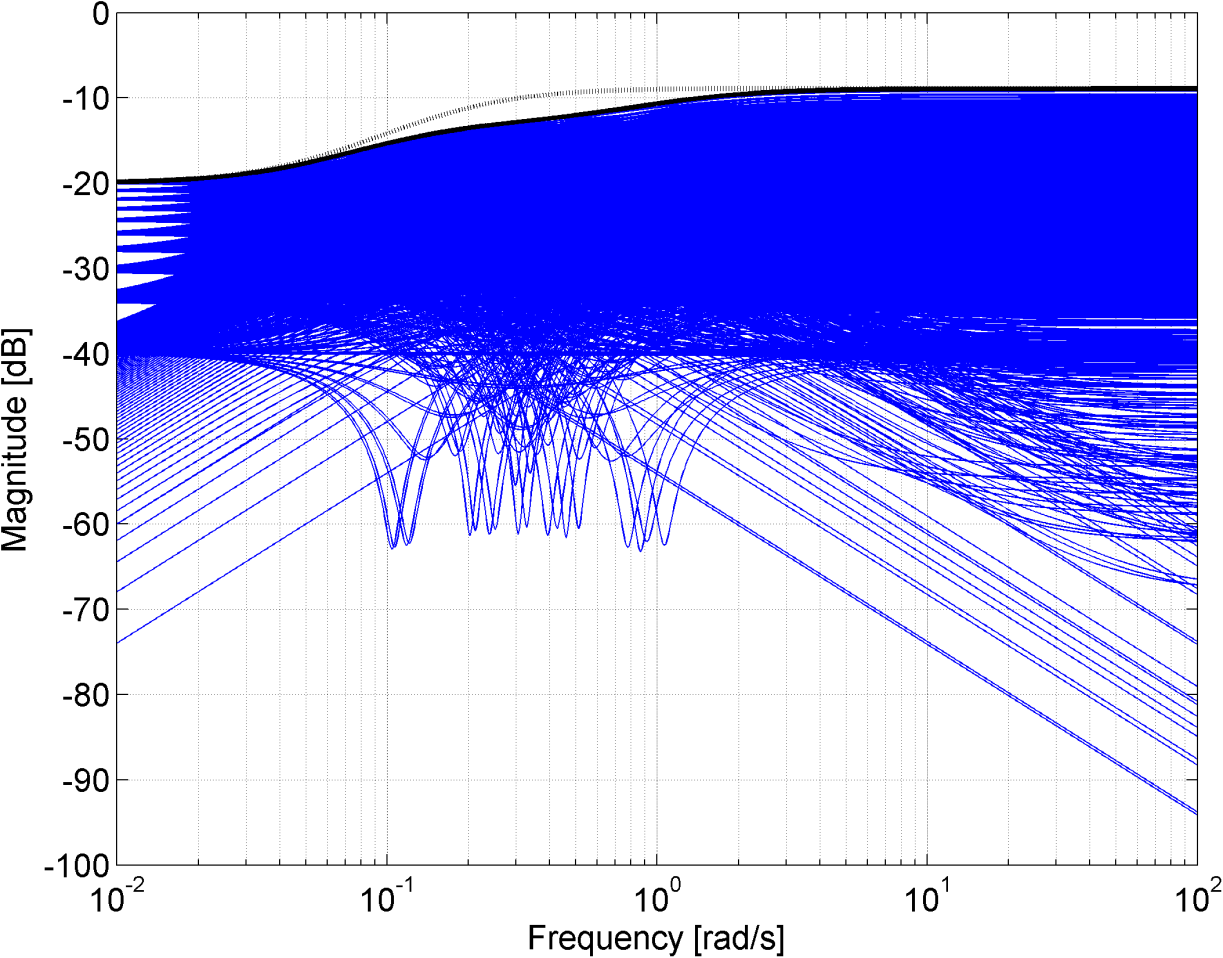
Bode magnitude plots–the set of normalized perturbations and weight functions W_M5_ (21) (dotted line) and W_M6_ (22) (solid line).

**Fig 8:**
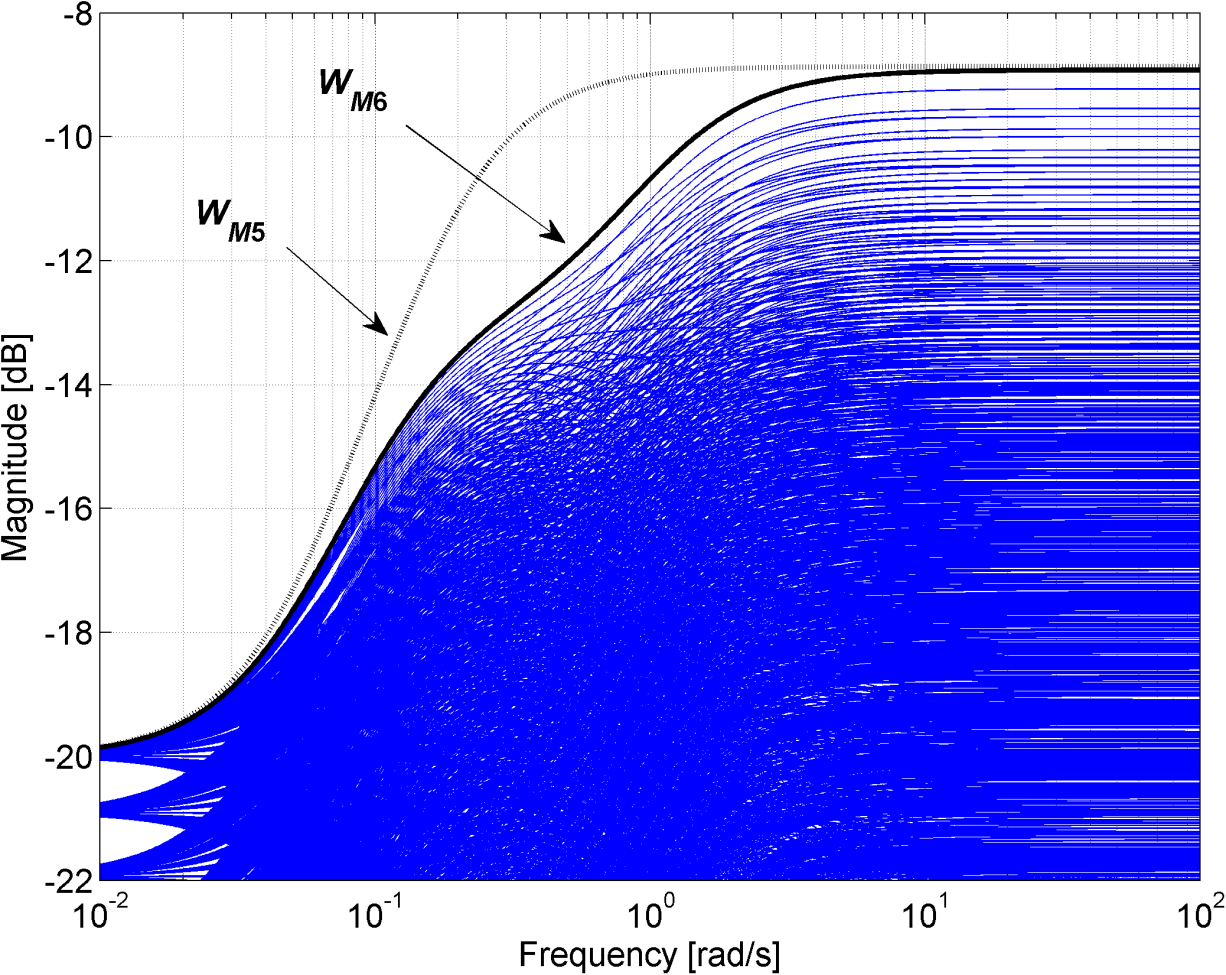
Zoomed version of Bode magnitude plots from Fig 7 –the set of normalized perturbations and weight functions W_M5_ (21) (dotted line) and W_M6_ (22) (solid line).

Figs 3, 5, 7 and 13 contain a great number of simulation results. They show the “representative” set of Bode magnitude plots of the normalized perturbations from (4) for all combinations of uncertain parameters with some selected steps (samples). Furthermore, they compare these sets with Bode magnitude plots of designed weights in order to visualize the preciseness of their coverage. Then, Figs 4, 6, 8 and 14 represent the zoomed versions of the full Figs 3, 5, 7 and 13, respectively, for a more detailed view.

### 4.1 First order time-delay plant

Initially, assume a frequently used model of a first order time-delay system described by the transfer function:

$$G(s)=\frac{K}{Ts+1}e^{-\Theta s}$$ (10)

where all three parameters (gain, time constant, and time-delay term) are supposed to be uncertain. In this example, all of them can vary within the interval *K*,*T*,Θ ∈ ⟨1,2⟩.

Recall that, instead of working directly with the parametric system, the model with unstructured multiplicative uncertainty is going to be created and used in the ensuing considerations.

The creation of the multiplicative model (1) consists in the selection of a nominal model *G*_0_(*s*) and a weight function *W*_*M*_(*s*).

Firstly, the nominal model is chosen by taking the mean values of the uncertain parameters i.e. this nominal model still contains the time-delay term:

$$G_{0}(s)=\frac{1.5}{1.5s+1}e^{-1.5s}$$ (11)

In the next step, the appropriate weight function, considered as the uncertainty envelope, has to be found in order to fulfill inequality (4) and cover from the upper side the normalized perturbation of even the worst possible case of uncertainty in the model (10). More approaches will be applied to this purpose.

The first weight function is determined on the basis of the equation from [29] which is also referred to in [14]:

$$W_{M}(s)=\frac{K_{\max}}{K_{0}}\cdot \frac{T_{0}s+1}{T_{\min}s+1}\cdot \frac{\tau s+1}{-\tau s+1}-1;\;\tau =\frac{\Theta_{\max}-\Theta_{\min}}{4}$$ (12)

where variables with “min” and “max” are the minimal or maximal possible values of the uncertain parameters from (10) and variables with “0” denote the mean parameter values from the nominal model in the form (11). In this example, the rule (12) leads to the weight function:

$$W_{M1}(s)=\frac{0.75s^{2}+1.58\bar{3}s+0.\bar{3}}{-0.25s^{2}+0.75s+1}$$ (13)

The potential problem with this weight (13) is that it is an unstable function and, according to [14], we always choose the weight to be stable and minimum phase in order to avoid unnecessary problems. Moreover, the weight function (13) approximates the normalized perturbations only at first sight–see Fig 3, where the comparison of Bode magnitude plots of designed weights is plotted together with a “representative” set of the normalized perturbations for all combinations of parameters with chosen steps *K* = 1:0.1:2, *T* = 1:0.1:2 and Θ = 1:0.1:2, and see especially its zoomed version in Fig 4 for a more detailed view. It is obvious that the weight *W*_*M*1_(*s*) (13) does not cover the normalized perturbations in the upper middle frequencies so it is inapplicable in the current form and it would have to be modified.

The second weight function is constructed by using the recommendation from [14] for unmodeled dynamics:

$$W_{M}(s)=\frac{\tau s+r_{0}}{\left(\tau /r_{\infty }\right)s+1}$$ (14)

where *r*_0_ is the relative uncertainty at steady-state (low frequencies), 1/*τ* is the approximate frequency at which the relative uncertainty reaches 100%, and *r*_∞_ is the magnitude of the weight at high frequencies. The suitable values for this example are *r*_0_ = 1/3, *τ* = 5, and *r*_∞_ = 3 and they result in:

$$W_{M2}(s)=\frac{5s+1/3}{\left(5/3\right)s+1}=\frac{5s+0.\bar{3}}{1.\bar{6}s+1}$$ (15)

Figs 3 and 4 show that the weight *W*_*M*2_(*s*) (15) is usable since it covers all of the normalized perturbations but it is rather conservative, with a relatively large distance to the curves of perturbations at lower and middle frequencies. However, approaching closer to the perturbations would mean that the weight would not cover all perturbations around the frequency where they reach 100% magnitude. Nevertheless, the “moved” weight can be multiplied by a correction factor in order to lift the gain slightly near the required frequency as advised in [14]. Thus, the weight (15) was “moved forward” by taking *τ* = 1.4 and subsequently lifted around *ω* = 6 by multiplication by second order function:

$$\begin{array}{l} W_{M3}(s)=\frac{1.4s+1/3}{\left(1.4/3\right)s+1}\cdot \frac{{\left(1/6\right)}^{2}s^{2}+1.06\left(1/6\right)s+1}{{\left(1/6\right)}^{2}s^{2}+\left(1/6\right)s+1}\\ =\frac{0.03\bar{8}s^{3}+0.2566s^{2}+1.45\bar{8}s+0.\bar{3}}{0.01296s^{3}+0.10\bar{5}s^{2}+1.6\bar{3}s+1} \end{array}$$ (16)

The good fit of the obtained weight *W*_*M*3_(*s*) (16) can be seen in Figs 3 and 4 as well.

Nonetheless, not only the weight function but also nominal model can be modified in order to derive a different model with unstructured multiplicative uncertainty. For example, it could be advantageous (because of facilitated controller design) to use a time-delay free nominal model and to consider the time-delay term as the uncertainty. So, the alternative nominal model for the example being studied can be:

$$G_{0}(s)=\frac{1.5}{1.5s+1}$$ (17)

Naturally, the set of normalized perturbations will differ from the previously plotted one. For this case, just one weight function is chosen in an analogical way as the function (16) was. The recommended weight can look like:

$$\begin{array}{l} W_{M4}(s)=\frac{3s+1/3}{\left(3/3\right)s+1}\cdot \frac{{\left(1/1.5\right)}^{2}s^{2}+1.15\left(1/1.5\right)s+1}{{\left(1/1.5\right)}^{2}s^{2}+\left(1/1.5\right)s+1}\\ =\frac{1.\bar{3}s^{3}+2.4481s^{2}+3.2\bar{5}s+0.\bar{3}}{0.\bar{4}s^{3}+1.\bar{1}s^{2}+1.\bar{6}s+1} \end{array}$$ (18)

A comparison of normalized perturbations and the weight (18) is depicted in Fig 5 while the detailed view of the same is provided by Fig 6.

### 4.2 Second order plant

In the next example, consider a second order system with two different time constants given by the transfer function:

$$G(s)=\frac{K}{\left(T_{1}s+1\right)\left(T_{2}s+1\right)}$$ (19)

where gain and time constants are supposed to lie within intervals *K* ∈ ⟨1.8,2.2⟩, *T*_1_ ∈ ⟨9,11⟩ and *T*_2_ ∈ ⟨0.9,1.1⟩.

The nominal model is chosen through the average values of the uncertain parameters, i.e.:

$$G_{0}(s)=\frac{2}{\left(10s+1\right)\left(s+1\right)}=\frac{0.2}{s^{2}+1.1s+0.1}$$ (20)

A first representative of possible weight functions can be found by using the recommendation for unmodeled dynamics (14). The appropriate values for this example are *r*_0_ = 0.1, *r*_∞_ = 0.36 and *τ* = 2 which leads to:

$$W_{M5}(s)=\frac{2s+0.1}{5.55s+1}$$ (21)

The obtained weight function (21) is of a low (first) order, but it is rather conservative as can be seen from Figs 7 and 8.

The selection of the second appropriate weight function is based on a simple idea. The worst possible case of uncertainty (which has to be covered by this weight function) in the model (19) is represented by just one plant with the greatest possible gain *K* = 2.2 and the shortest possible time constants *T*_1_ = 9 and *T*_2_ = 0.9. This combination of parameters directly corresponds to the “uppermost” magnitude characteristics of normalized perturbation from Figs 7 and 8. Thus, it can be easily derived that the worst case is covered exactly by the weight function:

$$W_{M6}(s)=\frac{2.9s^{2}+2.2s+0.1}{8.1s^{2}+9.9s+1}$$ (22)

Bode magnitude plots of both weight functions *W*_*M*5_ (21) and *W*_*M*6_ (22) together with the “representative” set of normalized perturbations are shown in Fig 7. The set of relative errors is plotted for all combinations of parameters with chosen steps *K* = 1.8:0.02:2.2, *T*_1_ = 9:0.2:11 and *T*_2_ = 0.9:0.02:1.1. The zoomed version of the same Bode magnitude plots is depicted in Fig 8.

Thus, if the second (exact) weight (22) is considered, the final model of the plant with unstructured multiplicative uncertainty is:

$$\begin{array}{l} G(s)=\left[1+W_{M6}(s)\Delta_{M}(s)\right]G_{0}(s)\\ {\left\|\Delta_{M}(s)\right\|}_{\infty }\leq 1\\ G_{0}(s)=\frac{0.2}{s^{2}+1.1s+0.1}\\ W_{M6}(s)=\frac{2.9s^{2}+2.2s+0.1}{8.1s^{2}+9.9s+1} \end{array}$$ (23)

This (second order plant) example also includes the robust stability analysis. For this purpose, a trio of PI controllers is supposed:

$$C_{1}(s)=\frac{0.5s+1}{s}$$ (24)

$$C_{2}(s)=\frac{2s+1}{s}$$ (25)

$$C_{3}(s)=\frac{s+1}{s}$$ (26)

and robust stability of the feedback loop with one of these controllers and plant family (23) is investigated successively.

**Fig 9:**
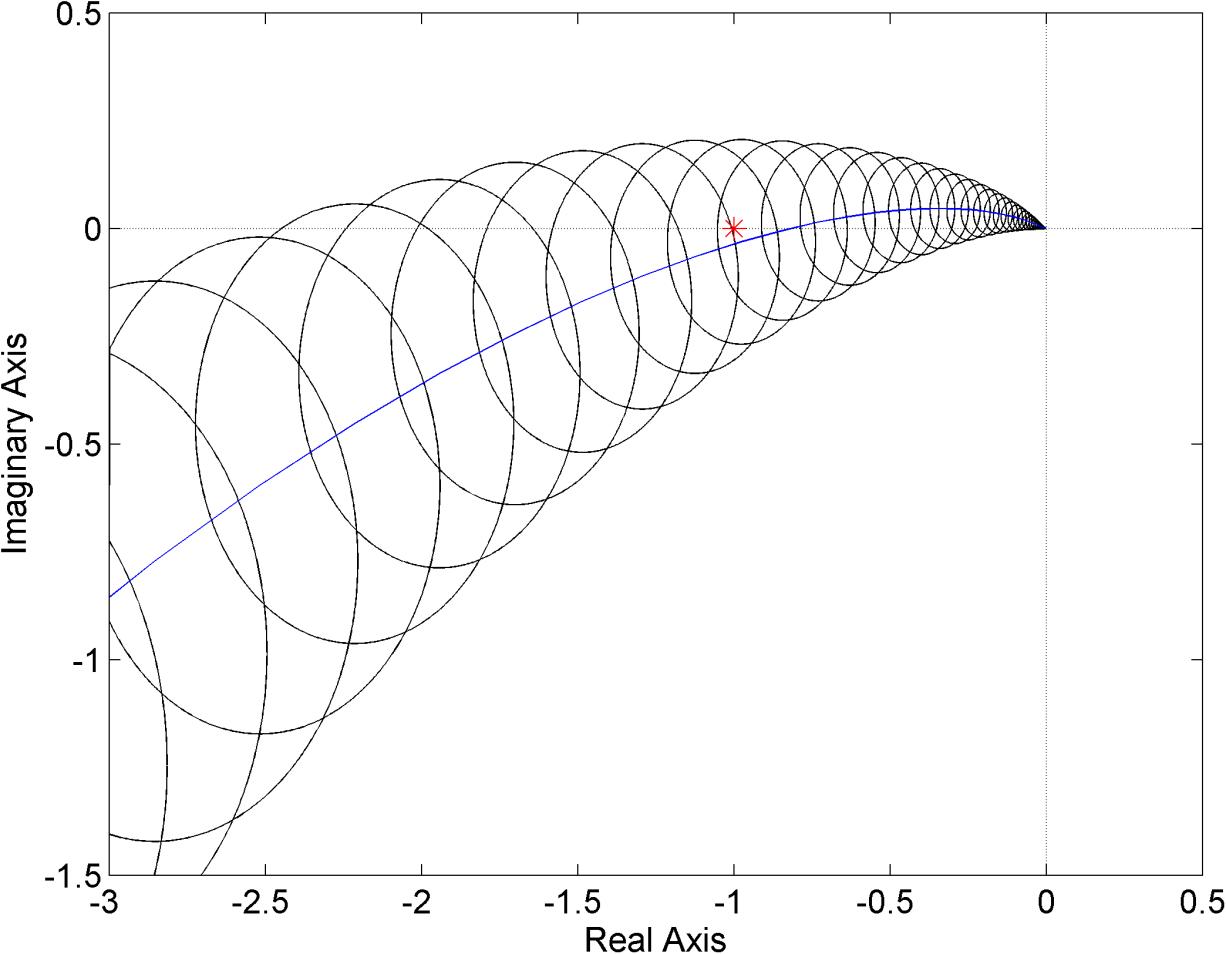
Envelope of Nyquist diagrams for the plant family (23) and controller (24)–a robustly unstable case.

For the first PI controller (24), the envelope of Nyquist diagrams given by circles with a radius of |*W*_*M*_(*jω*)*L*_0_(*jω*)| around the Nyquist diagram of the nominal *L*_0_(*jω*) (blue curve) is plotted in Fig 9. It can be clearly seen that the critical point [-1, 0*j*] is included in the envelope and consequently the closed loop with the controller (24) and family of systems (23) is robustly unstable.

**Fig 10:**
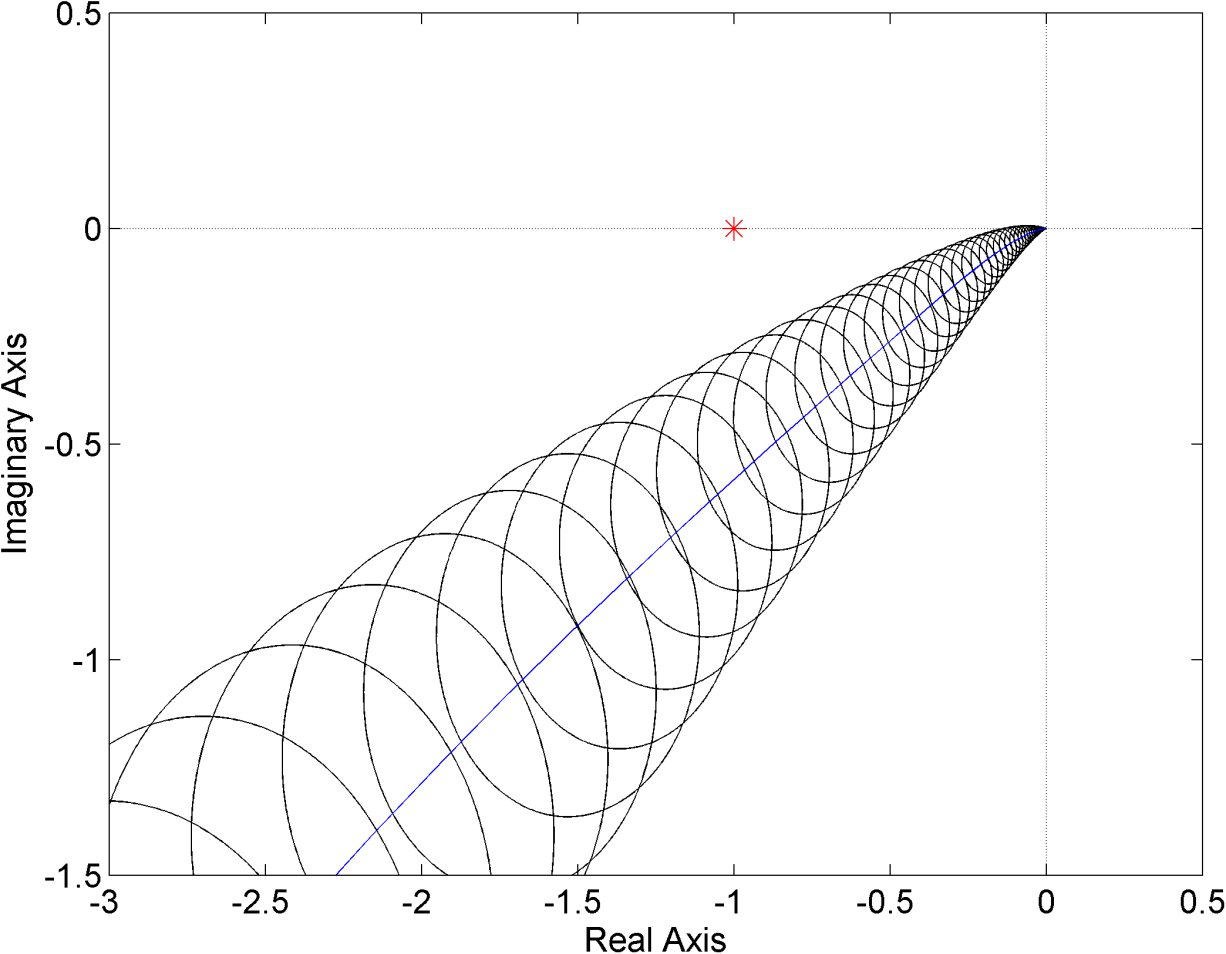
Envelope of Nyquist diagrams for the plant family (23) and controller (25)–a robustly stable case.

The similar envelope of Nyquist diagrams for the second controller (25) is shown in Fig 10. In this case, the critical point is excluded from the envelope which entails the robust stability of the closed loop with the plant family (23) and controller (25).

**Fig 11:**
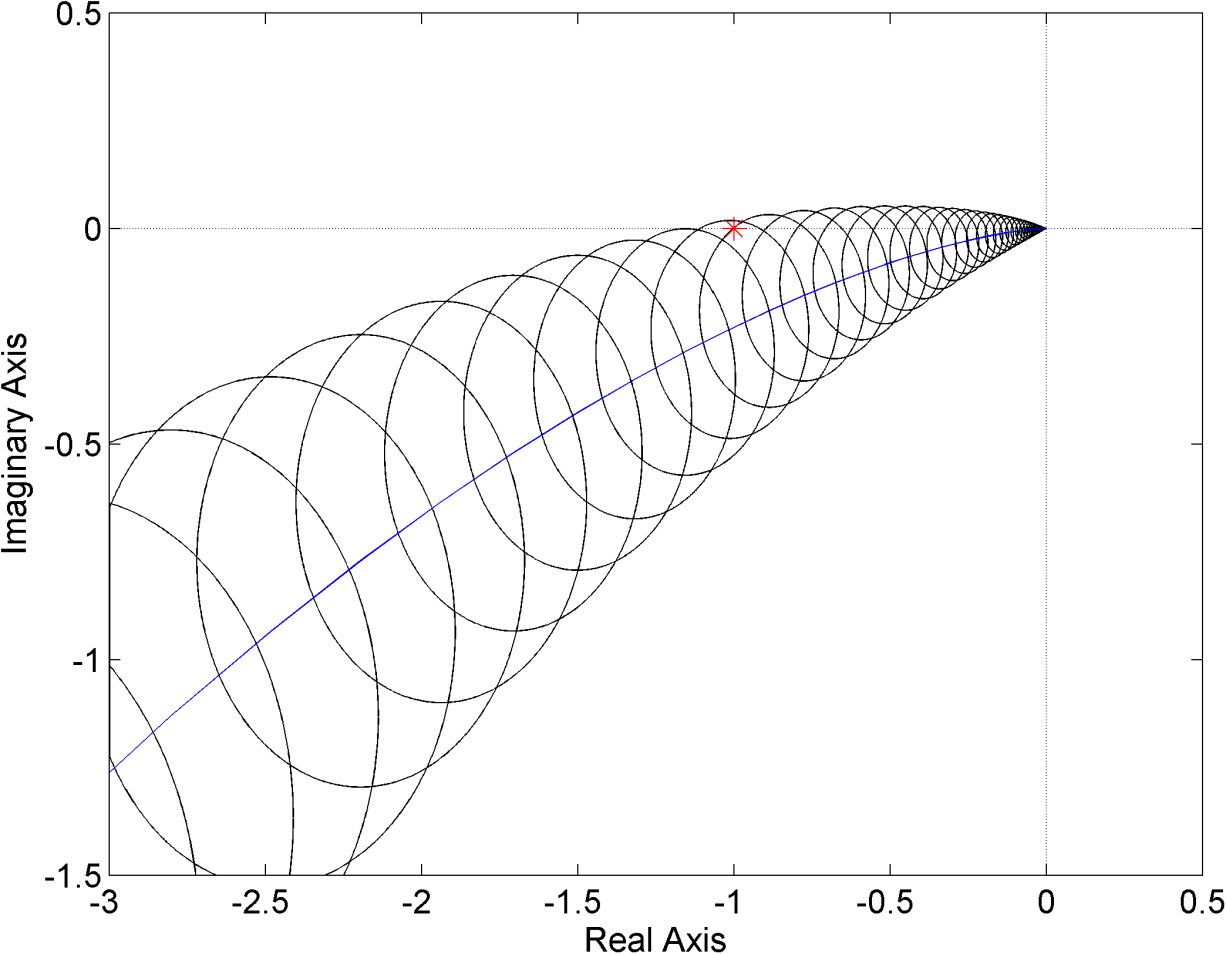
Envelope of Nyquist diagrams for the plant family (23) and controller (26)–a robustly unstable? case.

Finally, the robust stability condition is visualized for the last PI controller (26)–see Fig 11. Note that the point [-1, 0*j*] is included in the envelope and thus, the closed-loop system is not robustly stable. However, as can be easily verified, the closed-loop system which includes the original plant model (19) with parametric uncertainty is, in fact, robustly stable. The robust instability holds true for the constructed unstructured multiplicative uncertainty model (23), but not for the original parametric model (19). This means that even if the weight function (22) covers the normalized perturbations as tightly as possible (see Fig 8), the family of systems (23) can still contain some members which are not stabilized by the controller (26) because the perturbations satisfying |Δ_*M*_(*jω*)| ≤ 1 at all frequencies are supposed. Consequently, one should be aware of potential conservatism in the investigation of robust stability when a system with parametric uncertainty is modeled as a system with unstructured multiplicative uncertainty. In other words, the necessary and sufficient robust stability condition can change to only a sufficient one.

Obviously, the utilization of a simpler first order weight function (21) would mean an even higher level of conservatism in the robust stability analysis.

**Fig 12:**
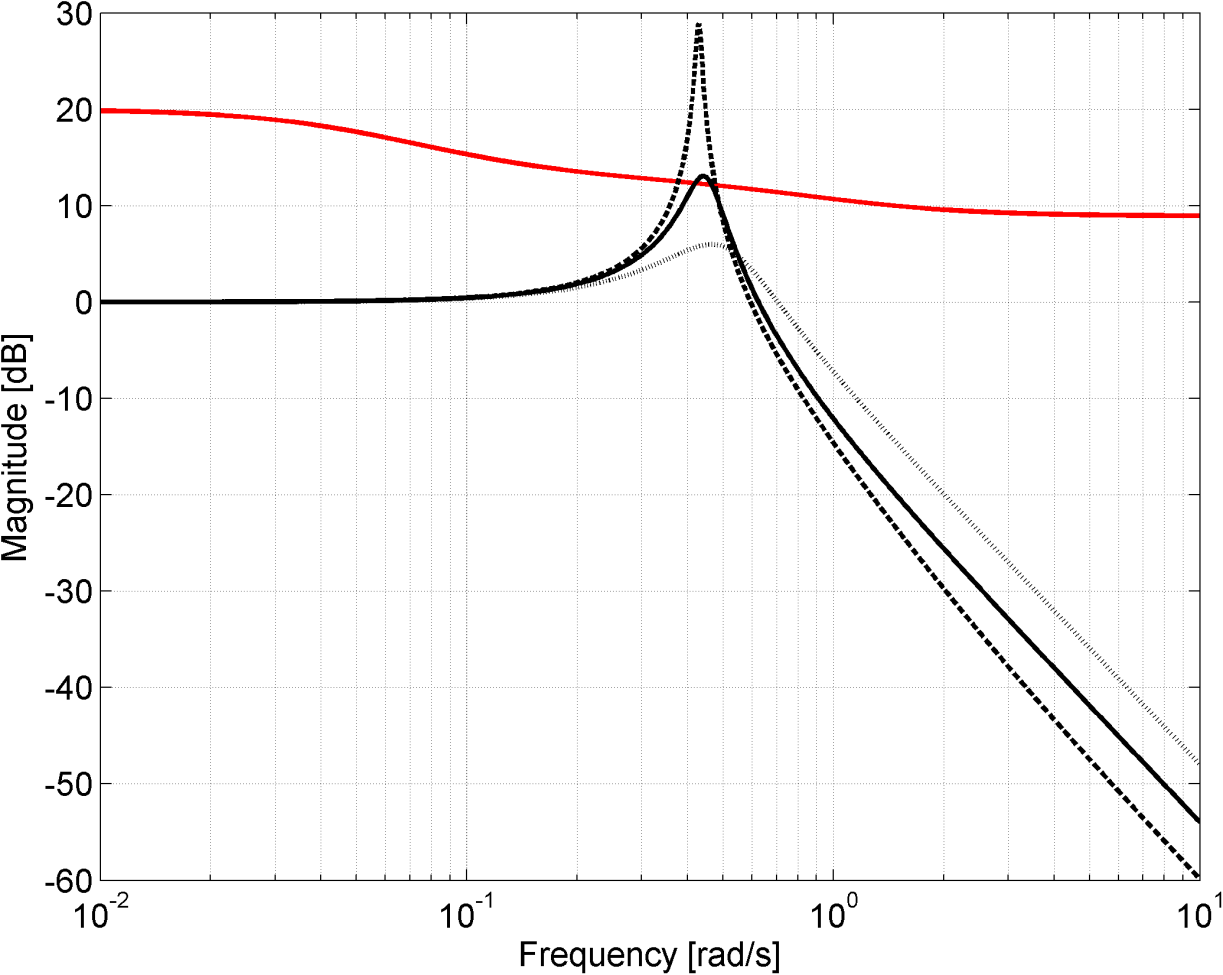
Visualization of robust stability condition in an alternative form (9)–the plant model (23) with controller (24) (dashed curve), (25) (dotted curve) or (26) (solid curve).

Alternatively, robust stability can be graphically tested and visualized by means of the condition in the form (9), i.e. the upper bound restricted Bode magnitude plots of complementary sensitivity functions can be used instead of the envelope of Nyquist diagrams. The plots for the loops with the model (23) and all three PI controllers (24)–(26) successively, and the upper bound 1/|*W*_*M*_(*jω*)| (red curve) are shown in Fig 12. As can be seen, the controller (24) (dashed curve) does not robustly stabilize the assumed model since the peak reaches beyond the upper bound. On the other hand, the second controller (25) (dotted curve) is robustly stabilizing one since the plot stays under the upper bound for all frequencies. And finally, the controller (26) (solid curve) is robustly non-stabilizing for the model (23) as the curve goes (slightly but still) beyond the upper bound. Naturally, the results obtained fully correspond to the envelopes of Nyquist diagrams previously plotted in Figs 9–11.

### 4.3 Third order plant

For the final case, assume a third order system (inspired by Burns [30]) with integrating behavior:

$$G(s)=\frac{1}{s\left(s^{2}+a_{2}s+a_{1}\right)}=\frac{1}{s^{3}+a_{2}s^{2}+a_{1}s}$$ (27)

where *a*_1_ ∈ ⟨3,5⟩ and *a*_2_ ∈ ⟨1,3⟩.

The nominal system is considered again as the transfer function with the mean values of the uncertain parameters from (27):

$$G_{0}(s)=\frac{1}{s^{3}+2s^{2}+4s}$$ (28)

The appropriate weight was initially chosen as:

$$W_{M7}(s)\approx \frac{0.35\left(s+1\right)}{{\left(0.52s\right)}^{2}+2\xi 0.52s+1}$$ (29)

with a damping ratio of *ξ* = 0.25. In this weight, the value 0.35 (in the numerator) agrees with the relative uncertainty at steady-state (low frequencies). Then the second order polynomial in the denominator means that the Bode plot starts to descend with the slope -40 dB/decade at frequency 0.52^−1^ rad/s (under the assumption of only a constant in the numerator) and that the Bode plot has a peak near this frequency. The magnitude of this peak depends on the size of a damping ratio. The lower damping ratio, the higher peak. However, the requested “final” slope at the high frequencies is only -20 dB/decade and thus the influence of the second order denominator is compensated by the first order polynomial in the numerator. Moreover, this term contributes to the Bode plot rise from the frequency 1 rad/s, i.e. near the beginning of the peak. The final weight function, after slight manual adjustment, has the form:

$$W_{M7}(s)=\frac{0.35s+0.35}{0.273s^{2}+0.26s+1}$$ (30)

**Fig 13:**
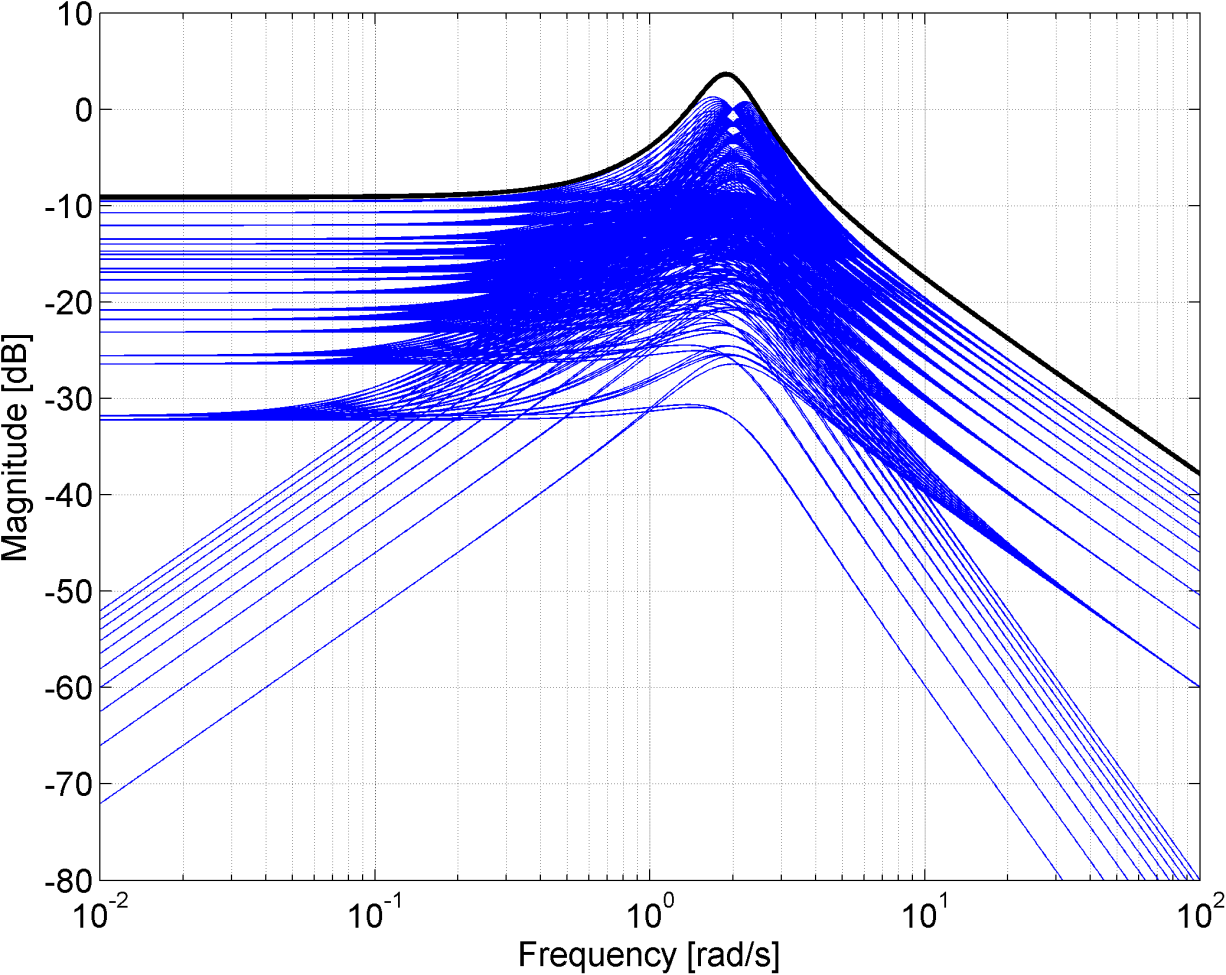
Bode magnitude plots–the set of normalized perturbations and weight function W_M7_ (30).

**Fig 14:**
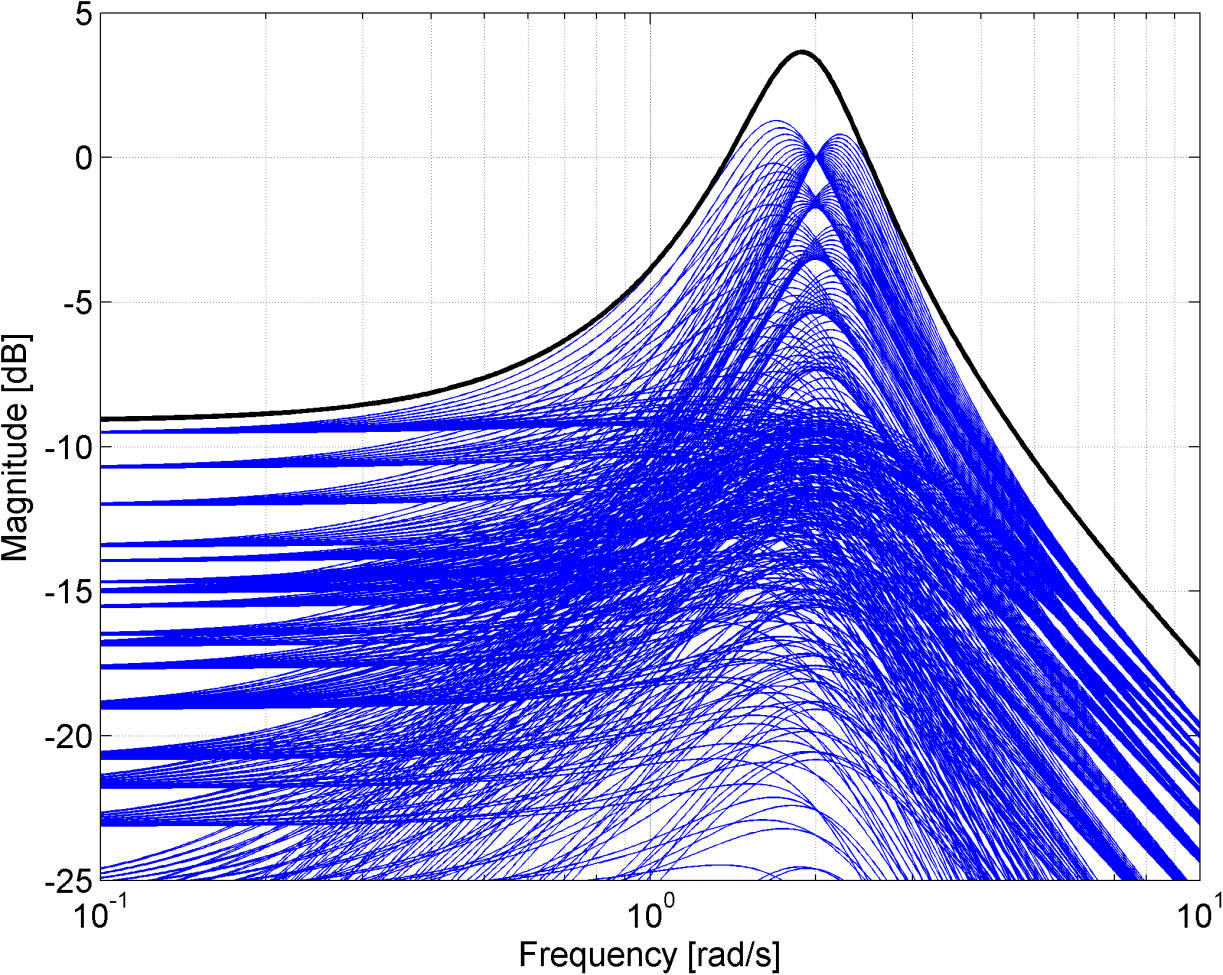
Zoomed version of Bode magnitude plots from Fig 13 –the set of normalized perturbations and weight function W_M7_ (30).

The Bode magnitude plots of normalized perturbations for all combinations of parameters according to *a*_1_ = 3:0.1:5, *a*_2_ = 1:0.1:3 together with the Bode magnitude plot of the weight function (30) are depicted in Fig 13. Then, the zoomed version (for both frequency and magnitude axis) of the same plots is shown in Fig 14.

So, the final model for the third example is:

$$\begin{array}{l} G(s)=\left[1+W_{M7}(s)\Delta_{M}(s)\right]G_{0}(s)\\ {\left\|\Delta_{M}(s)\right\|}_{\infty }\leq 1\\ G_{0}(s)=\frac{1}{s^{3}+2s^{2}+4s}\\ W_{M7}(s)=\frac{0.35s+0.35}{0.273s^{2}+0.26s+1} \end{array}$$ (31)

**Fig 15:**
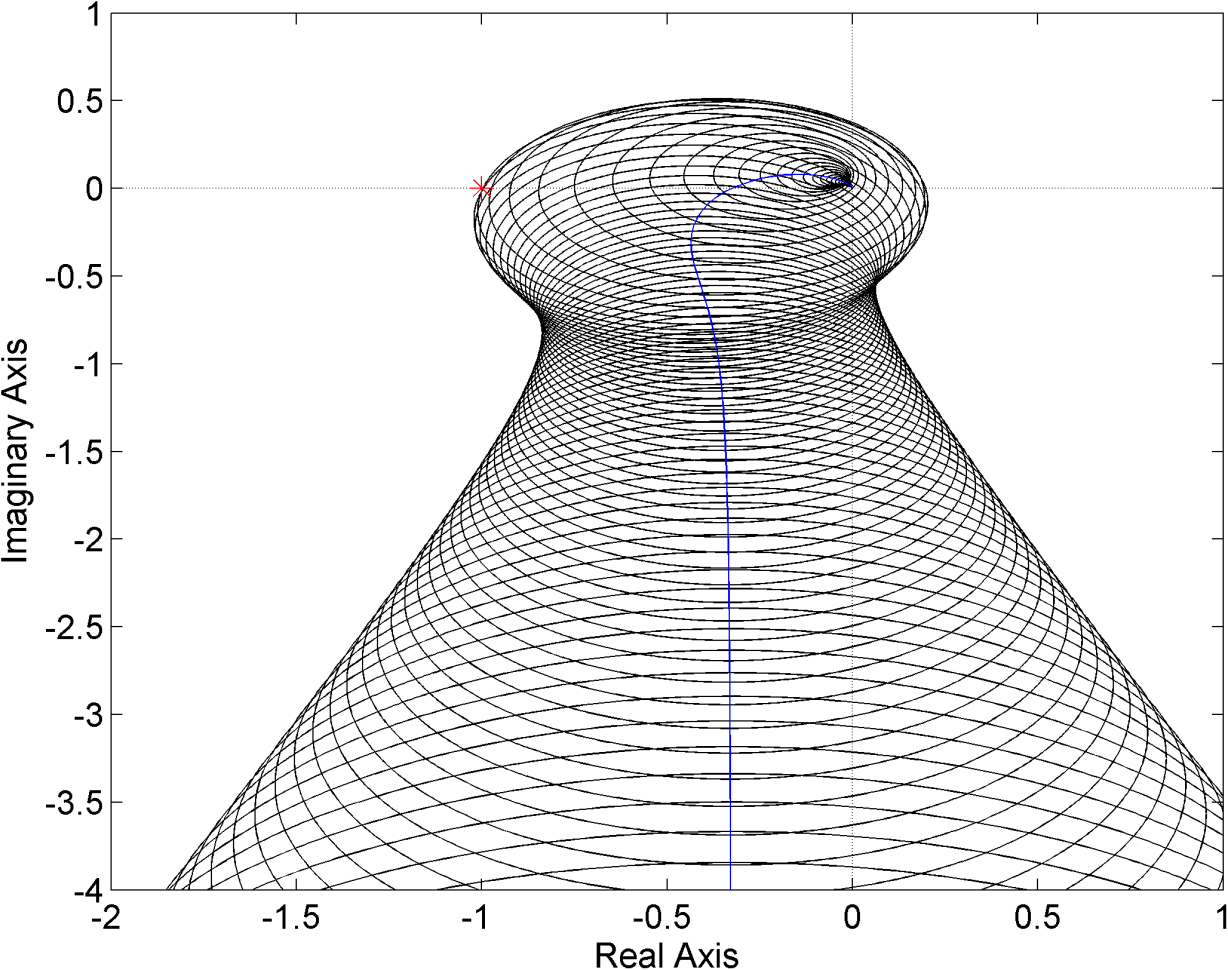
Envelope of Nyquist diagrams close to the robust stability border–the plant family (31) and P controller with gain 2.61.

The plant family (31) is supposed to be connected in the feedback loop with a simple proportional controller and the next aim of this example is to find a critical gain of this controller which brings the feedback system to the robust stability border. The envelope of Nyquist diagrams for the gain 2.61 is plotted in Fig 15. As can be seen, this envelope “touches” the critical point which means the feedback control system with the proportional controller gain near the value 2.61 is on the robust stability border. In fact, the critical gain for the original parametrically uncertain system (27) is 3 (due to the conservatism discussed in the previous example).

## 5. Conclusion

This article focused on the modeling and robust stability analysis of continuous-time LTI SISO systems with unstructured multiplicative uncertainty. The examples presented herein have shown the techniques for the construction of multiplicative uncertainty models from systems with parametric uncertainty via the selection of suitable nominal models and weight functions. Moreover, the robust stability of the feedback control loops that contain multiplicative uncertainty plants was analyzed and their conservatism in comparison with the usage of “original” parametric uncertainty plants was discussed.
